# Anti-Viral Activity of Conessine Against Influenza A Virus

**DOI:** 10.3390/ijms26157572

**Published:** 2025-08-05

**Authors:** Won-Kyung Cho, Jin Yeul Ma

**Affiliations:** Korean Medicine (KM) Application Center, Korea Institute of Oriental Medicine, 70 Cheomdan-ro, Dong-gu, Daegu 41062, Republic of Korea

**Keywords:** conessine, influenza A virus, antiviral, viral attachment, hemagglutinin

## Abstract

Conessine is a steroidal alkaloid found in many plants. The pharmacological efficacies of conessine on various ailments, including antiviral effects against Zika, Herpes, and Coronavirus, were reported. However, the effect of conessine on the influenza virus was still unknown. In this study, conessine exhibited a strong inhibitory effect against influenza A virus (IAV) infection. We examined the effect of conessine on IAV using green fluorescent protein (GFP)-expressing Influenza A/PR8/34 and wild-type A/PR8/34. The fluorescence-activated cell sorting, fluorescence microscopy, cytopathic effect analysis, and plaque assay demonstrated that conessine significantly inhibits IAV infection. Consistently, immunofluorescence results showed that conessine strongly reduces the expression of IAV proteins. The time-of-drug-addition assay revealed that conessine could affect the viral attachment and entry into the cells upon IAV infection. Further, conessine eradicated the virus before binding to the cells in the early stage of viral infection. Our results suggest that conessine has strong anti-viral efficacy against IAV infection and could be developed as an anti-influenza viral agent.

## 1. Introduction

Influenza viruses cause respiratory and systemic symptoms such as headache, cough, fever, sore throat, and muscular pain, and can lead to death in high-risk individuals [1]. Influenza A viruses (IAV) are the leading cause of the annual seasonal flu epidemics [2]. Frequent antigenic point mutations and shifts during RNA replication generate new influenza A variants that differ in neuraminidase (NA) and/or hemagglutinin (HA) [3]. Therefore, developing a perfect vaccine in advance for emerging new influenza variants is impossible. Influenza viruses have a negative-sense single-stranded RNA genome and belong to the Orthomyxoviridae family [2]. Influenza virus has Nonstructural (NS) 1, Nucleoprotein (NP), Matrix protein 1, 2 (M1, M2), and RNA polymerase components (PA, PB1, and PB2), as well as HA and NA proteins [4]. Antiviral agents against the influenza virus are classified according to the target protein they inhibit. Currently, NA inhibitors such as oseltamivir, zanamivir, and peramivir, and PA inhibitors such as baloxavir marboxil are used clinically. However, various side effects and drug-resistant strains against them have appeared, and the discovery of new anti-influenza viral agents is urgent [5,6].

Conessine is a steroidal alkaloid found in many plants, including *Holarrhena antidysenterica*, *Holarrhena floribunda*, and *Funtumia elastica*. Conessine has been reported to have various pharmacological effects, such as anti-plasmoidal [7], anti-amebiasis [8,9], anti-tumor [10], anti-muscular atrophy [11], and anti-acetylcholinesterase [12]. Several recent studies demonstrated that conessine has anti-viral effects against the ZIKA virus [13], human coronaviruses (HCoV-OC43, HCoV-NL63, and MERS-CoV) [14], Dengue virus [15], Vesicular stomatitis virus [15], and Herpes simplex virus [15]. In the present study, we discovered for the first time that conessine inhibits influenza A virus infection via blockage of viral binding and entry, and exerts a virus-eradicating effect.

## 2. Results

### 2.1. Cytotoxicity of Conessine

**Figure 1:**
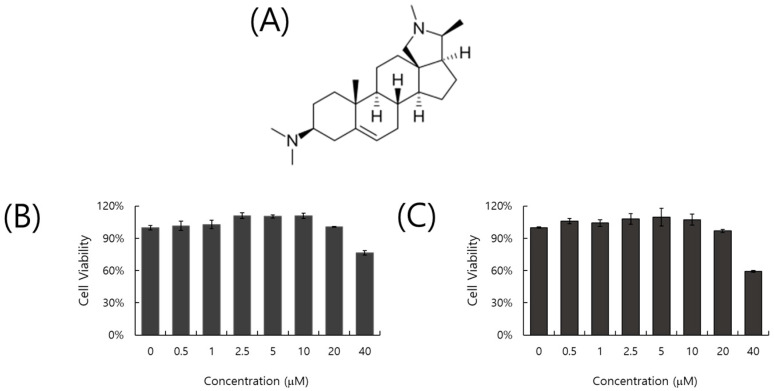
Chemical structure (**A**) and cytotoxicity of conessine on A549 (**B**) and RAW 264.7 (**C**) cells. Conessine was serially diluted from 40 to 0.5 µM and treated in each cell for 24 h at 37 °C. CCK-8 reagent was added to evaluate the toxicity of conessine in the cells. The data were presented as the mean ± standard deviation based on three replicates in three different experiments.

The toxicity of conessine on the A549 and RAW 264.7 cells was examined using a CCK-8 assay. As shown in Figure 1B,C, conessine did not affect cell viability up to 20 µM (Figure 1B,C). The CC50 values of conessine on A549 and RAW 264.7 cells were 62.32 and 46.39 µM, respectively.

### 2.2. Conessine Exhibits an Anti-Viral Effect Against the Influenza A Virus

**Figure 2:**
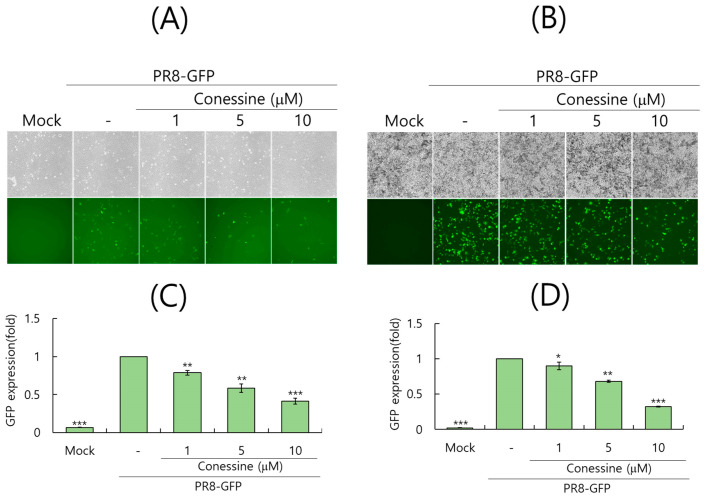
Antiviral effect of conessine against PR8-GFP IAV infection. (**A**–**D**) PR8-GFP IAV (10 MOI) and conessine (0, 1, 5, or 10 μM) were mixed and preincubated for 1 h at 4 °C. The mixtures were added to A549 (**A**,**C**) or RAW 264.7 (**B**,**D**) cells for 2 h at 37 °C. After washing, the cells were further incubated for 24 h at 37 °C. The brightfield and fluorescence images were obtained using a fluorescence microscope with 200× magnification (**A**,**B**). The cells were FACS-analyzed to compare each group’s relative GFP expression levels. The data represent the mean ± standard deviation based on three independent experiments (**C**,**D**). Statistical significance was assessed via an unpaired Student’s *T*-test. *** *p* < 0.0005, ** *p* < 0.005, * *p* < 0.05 compared to the PR8-GFP IAV infected group.

To investigate the effect of conessine on influenza A virus infection, we used green fluorescence protein-expressing Influenza A/PR8/34 (PR8-GFP IAV) virus. A549 cells and RAW 264.7 cells were infected with PR8-GFP IAV and conessine (0, 1, 5, or 10 µM) for 24 h. The fluorescence microscopy results showed that conessine significantly reduced IAV-induced GFP expression in both cells, in a dose-dependent manner (Figure 2A,B). Next, we performed FACS analysis on PR8-GFP IAV-infected cells in the presence or absence of conessin to compare the level of GFP expression and confirm the antiviral efficacy of conessin. As shown in Figure 2C,D, conessine dose-dependently repressed IAV-induced GFP expression in A549 and RAW 264.7 cells, with 50% inhibitory concentration (IC50) of 7.02 µM and 7.4 µM, respectively. SI index of conessine in A549 and RAW 264.7 cells was determined as 8.83 and 6.26.

**Figure 3:**
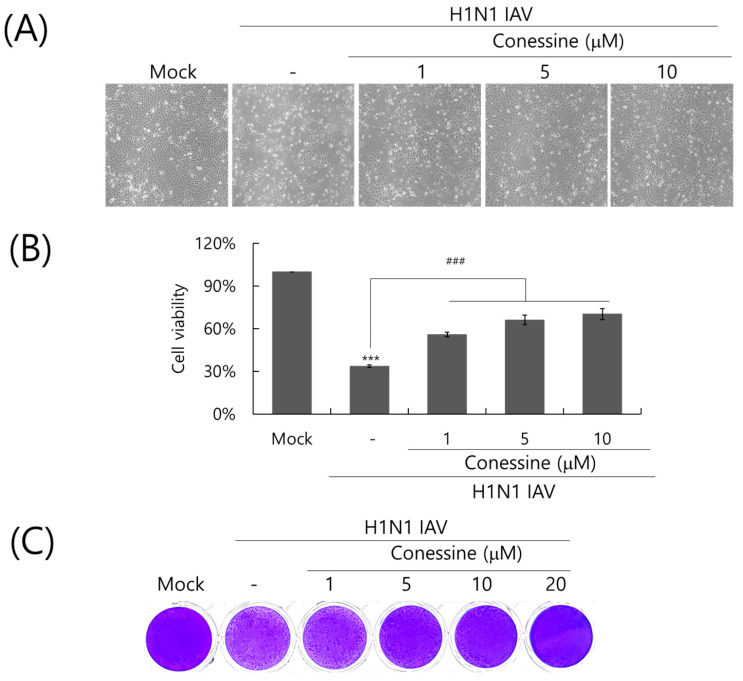
Antiviral effect of conessine against H1N1 IAV infection. (**A**,**B**) Conessine (0, 1, 5, or 10 μM) and 10 MOI H1N1 IAV were mixed and cotreated to A549 cells. At 48 h post-infection, the cells were captured with a brightfield microscope with 200× magnification (**A**). Cell viability was assessed by CCK-8 assay (**B**). (**C**) Conessine at 0, 1, 5, 10, or 20 μM and H1N1 IAV were cotreated to MDCK cells. After washing with PBS to remove the uninfected virus and conessine, the cells were overlaid with 1.5% agarose-containing DMEM and further incubated for 72 h. The cells were fixed with paraformaldehyde and stained with 1% crystal violet. The data were presented as the mean ± standard deviation based on three independent experiments. Statistical significance was assessed via an unpaired Student’s *T*-test. *** *p* < 0.0005 compared with the virus-infected group and ^###^ *p* < 0.0005 compared with uninfected mock group.

Next, we investigated the impact of conessine on the cytopathic effects induced by wild-type H1N1 IAV. We compared the cell viability at three concentrations of conessine treated in the presence of H1N1 IAV. Consistent with Figure 2, conessine significantly increased cell viability by blocking the cytopathic effect of IAV infection in a dose-dependent manner (Figure 2A,B). In the absence of conessine, the cell viability of IAV-infected cells was 30%. However, 1, 5, and 10 µg of connessine increased the cell viability decreased by IAV infection to more than 50, 60, and 70%, compared with the virus-infected control, respectively. Additionally, we confirmed the anti-influenza A virus effect of conessine using a plaque assay. When H1N1 IAV without or with conessine (1, 5, 10, or 20 μM) was infected into MDCK cells, conessine from 5 μM drastically inhibited Influenza virus plaque formation (Figure 3C). These results support that conessine has a significant anti-influenza A viral effect.

### 2.3. The Effect of Conessine on Viral Proteins in IAV-Infected Cells

**Figure 4:**
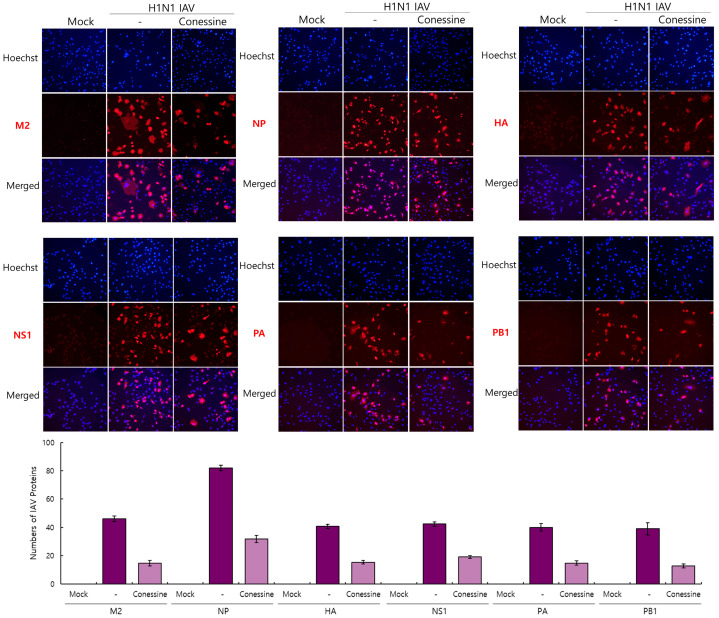
Effect of conessine on IAV M2, NP, HA, NS1, PA, and PB1 protein expression. Conessine (5 μM) and 10 MOI H1N1 IAV were cotreated with A549 cells. The cells were immuno-stained with antibodies specific for influenza viral proteins at 24 h post-infection. Hoechst 33342 was used to stain the nuclei of the cells. The red viral proteins and blue nuclei were detected under a fluorescent microscope with 200× magnification.

To confirm the effect of conessine on IAV infection, we performed an Immunofluorescence analysis on viral proteins, such as M2, NP, HA, NS1, PA, and PB1, in A549 cells infected with H1N1 IAV in the presence or absence of conessine. At 24 h post-infection, the cells were fixed with paraformaldehyde and incubated with specific antibodies against IAV proteins. The red fluorescent Alexa 594-tagged antibodies were added to detect viral proteins, followed by Hoechst 33342 to detect nuclei. Consistent with Figure 2 and Figure 3, in the presence of conessine, all viral proteins were significantly repressed (Figure 4).

### 2.4. Conessine Prevents IAV Binding and Entry to the Cells and Shows Direct Virucidal Effects

**Figure 5:**
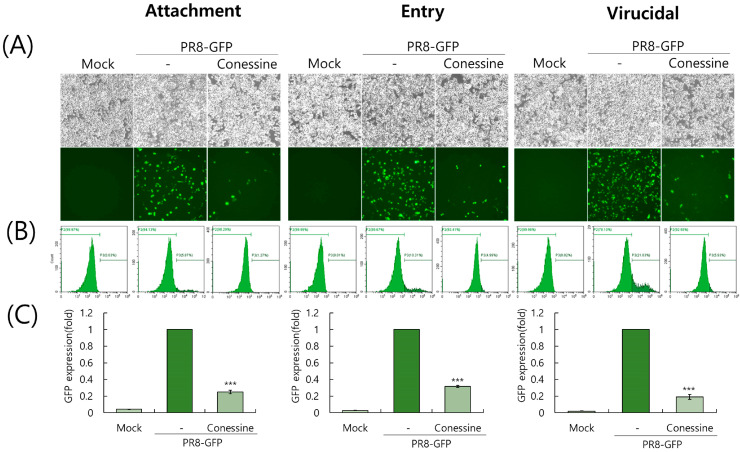
Conessine inhibits IAV infection at the early stages. The time-of-addition experiment was conducted under different incubation times and temperature conditions for infecting cells with conessine and PR8-GFP IAV, as described in the Section 4 in detail. (**A**) The cell images were obtained using fluorescence microscopy with 200× magnification (**B**,**C**). The GFP-expressing cells were counted by flow cytometry. The data represent the mean ± standard deviation based on three independent experiments. Statistical significance was assessed via an unpaired Student’s *t*-test. *** *p* < 0.005 compared to the virus-infected group.

Using different incubation conditions of virus and conessine, we investigated whether conessine could influence the viral binding and entry upon infection, and exert direct virus eradication before binding to the cells. Since influenza viruses could bind to the cells, but not penetrate the cells at 4 °C, we checked the effect of conessine on IAV binding and entry into the cells. To examine the virucidal effect of conessine, conessine and IAV were co-incubated for 1 h before infection of the cells. Fluorescence microscopy (Figure 5A) and flow cytometry (Figure 5B,C) results showed that conessine strongly inhibited influenza viral attachment and entry, and exhibited a potent virucidal effect. These results suggest that conessine has a strong anti-influenza viral infection by preventing viral infection at an early stage.

### 2.5. Anti-Influenza Viral Effect of Conessine Through Hemagglutination Inhibition

**Figure 6:**
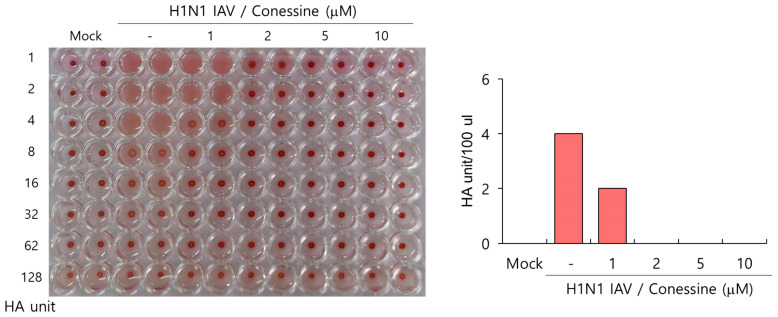
Conessine blocks IAV-induced hemagglutination. Conessine (0, 1, 2, 5, or 10 µM) and H1N1 IAV mixture were incubated with 1% RBCs. RBCs in the absence of viruses were aggregated. RBCs in the virus-infected well were hemolyzed. HA titers were calculated as HA units/100 µL compared to the virus control.

Because conessine has a strong inhibitory effect on IAV binding and entry into the cells, as demonstrated in Figure 5, we examined whether conessine could affect IAV-induced hemagglutination. The HA protein of IAV attaches to the sialic acid on the cell membrane and induces hemagglutination of red blood cells. HA is an essential protein for IAV binding and entry into the cells. When we checked the effect of conessine on hemagglutination by IAV infection, conessine dose-dependently repressed the hemagglutination (Figure 6). H1N1 virus without conessine caused hemagglutination and showed 4 HA units, while 1 µM conessine reduced HA units by 2-fold, and 2 µM completely inhibited RBC aggregation. These results indicate that conessine significantly blocks the HA protein, preventing viral binding and cell entry.

### 2.6. Effect of Conessine on Neuraminidase Activity

**Figure 7:**
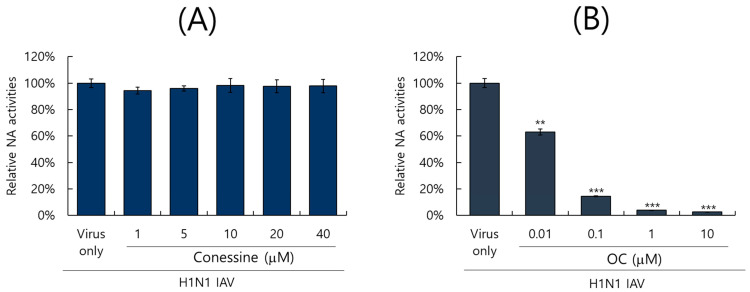
Effect of conessine on IAV neuraminidase activity. Conessine (**A**) or oseltamivir carboxylate (**B**) at the indicated concentrations was mixed with H1N1 IAV and subjected to a neuraminidase inhibition assay according to the manufacturer’s instructions. The NA activities were detected at an excitation wavelength of 365 nm and an emission wavelength of 445 nm. The data represent the mean ± standard deviation based on three independent experiments. Statistical significance was assessed via an unpaired Student’s *T*-test. *** *p* < 0.0005, ** *p* < 0.005 compared to the virus-infected group.

Neuraminidase of IAV facilitates the progeny release from the infected cell at a late stage of infection and is an important target for anti-influenza virus drug development [16]. In this regard, we tested whether conessine could inhibit the NA activity of IAV using the NA-Fluor Influenza Neuraminidase assay kit. Serially diluted conessine or oseltamivir carboxylate, as a positive control, was mixed with H1N1 IAV, and the reaction was performed according to the manufacturer’s protocol. As presented in Figure 7A, conessine did not exhibit anti-neuraminidase activity. In contrast, oseltamivir carboxylate exerted a strong inhibitory effect on the NA of H1N1 IAV (Figure 7B). These results imply that conessine did not block IAV progeny release after propagation in the late stage of IAV infection.

## 3. Discussion

Influenza viruses mutate every year, making vaccine development difficult. It is necessary to discover new antiviral candidates to overcome the shortcomings of current antivirals in clinical use. In this study, we examined the effect of conessine against influenza virus infection in A549 lung cells and RAW 264.7 macrophages, using the GFP-expressing influenza A virus and wild-type H1N1 influenza viruses. Conessine significantly dose-dependently repressed the GFP expression by GFP-IAV infection in both RAW 264.7 macrophages and A549 lung cells, and inhibited the cytopathic effect and plaque formation induced by H1N1 IAV infection in A549 cells. To clarify which stages of IAV infection are affected by conessine, we performed the time-of-drug-addition assay using different incubation temperatures and times of conessine and IAV. FACS analysis and fluorescent microscopy results showed that conessine potently prevents viral attachment and penetration into the cells in the early stage of infection. In particular, conessine significantly eradicated the influenza virus before it binds to cells upon infection. Influenza virus infection begins when the viral envelope protein, hemagglutinin, binds to a sialic acid-linked glycoprotein receptor on the host cell [17]. Several research studies were conducted to discover novel anti-influenza viral agents by blocking the binding of sialic acid to HA or by inhibiting HA. The pentacyclic triterpenes [18] and their derivatives [19] have been reported to inhibit IAV infection by blocking viral binding and HA activity. Chang YJ et al. have screened sialic acid inhibitors targeting HA using computer modeling to find new anti-influenza viral agents [20]. Aureonitol, which is found in Fungi, was reported to inhibit IAV infection by modulation of HA, not affecting NA activity [21]. Neoechinulin B [22], Isoquercitrin [23], and Amentoflavone [17], present in natural products, have been reported as anti-influenza virus agents via the inhibition of both NA and HA proteins of IAV. Hemagglutination inhibition assay confirmed the inhibitory effect of conessine on Hemagglutination by IAV infection. These results indicate that the potent antiviral effect of conessine against IAV infection is closely related to inhibition of the HA protein, thereby inhibiting the binding of the virus to cells. Although conessine had no inhibitory effect on the neuraminidase activity of IAV, it potently prevented IAV infection in the early stage of infection through HA modulation and virus eradication. We have demonstrated for the first time that conessine has an inhibitory effect on the influenza virus, and it is considered that it has the potential to be developed into an antiviral agent through combined treatment with NA inhibitors.

## 4. Materials and Methods

### 4.1. Compound, Cell Culture, and Viruses

Conessine (PubChem CID 441082) was purchased from Sigma-Aldrich (St. Louis, MO, USA). Murine macrophage RAW 264.7 (TIB-71) and human lung A549 cells (CCL-185) were cultured in RPMI 1640 medium (Hyclone, Logan, UT, USA), and Madin Darby Canine Kidney cells (MDCK, CCL-34) (ATCC, Manassas, VA, USA)were maintained in EMEM medium with 10% fetal bovine serum and penicillin and streptomycin (100 U/mL) at 37 °C with 5% CO_2._ Influenza A/PR8/34 (PR8-GFP) and A/PR8/34 (H1N1) viruses were received from Dr. Jong-Soo Lee (Chungnam National University, Daejeon, Republic of Korea). HBPV-VR-32 (H3N2) influenza virus was purchased from the Korea Bank for Pathogenic Viruses (KBPV). The viruses were amplified using a 10-day-old chicken embryo. All virus-related experiments were conducted under Biosafety Level 2.

### 4.2. Cytotoxicity Assay

A549 cells (5 × 10^4^ cells/well) and RAW 264.7 cells (1 × 10^5^ cells/well) were seeded in 96 wells and incubated for 24 h at 37 °C. Conessine (1 to 40 µM) was added to the cells for 24 h. The cells were treated with 10 μL of CCK-8 reagent (Dojindo, Rockville, MD, USA) for 2 h. Absorbance at a wavelength of 450 nm was detected using a microplate reader (Promega, Madison, WI, USA).

### 4.3. Cotreatment Assay of Virus and Conessine

Influenza viruses PR8-GFP or PR8 (H1N1) IAV (10 MOI) and conessine at the indicated concentrations were co-incubated for 1 h at 4 °C. The mixtures were added to A549 or RAW 264.7 cells for 2 h at 37 °C. After washing with PBS, the cells were further incubated for GFP expression or cytopathic effect formation.

### 4.4. Flow Cytometry

The cells infected with 10 MOI PR8-GFP IAV in the presence of conessine at 0, 1, 5, or 10 µM were harvested in PBS. The cells were washed and fixed with 4% paraformaldehyde for 10 min at room temperature. The cells were resuspended in PBS and analyzed using a CytoPLEX flow cell counter (Beckman Coulter Inc., Pasadena, CA, USA).

### 4.5. Cytopathic Effect Inhibition Assay

The mixtures of H1N1 IAV (10 MOI) and conessine (0, 1, 5, or 10 µM), preincubated for 1 h at 4 °C, were added to A549 cells for 2 h at 37 °C. The cells were washed with PBS and further incubated for 48 h. The cell viability was determined using a CCK-8 assay.

### 4.6. Plaque Inhibition Assay

MDCK cells (5 × 10^5^ cells/well) were seeded in 12-well plates. H1N1 IAV and 0, 1, 5, 10, or 20 µM conessine were mixed at 4 °C for 1 h. The mixtures were cotreated with MDCK cells for 1 h at 37 °C. The cells were washed with PBS, overlaid with a 1.5% agar-containing DMEM medium, and incubated for 72 h. After fixing with 4% paraformaldehyde for 10 min, the agar overlay was removed, and the cells were crystal violet-stained.

### 4.7. Time-of-Drug-Addition Assay

For an attachment step, 10 MOI of PR8-GFP IAV and 10 µM conessine were cotreated to RAW 264.7 cells for 30 min at 4 °C. The cells were washed with PBS and incubated for 24 h at 37 °C. For an entry step, the cells were infected with PR8-GFP IAV for 30 min at 4 °C. Conessine was added to the cells and incubated for 30 min at 37 °C. After washing, the cells were further incubated for 24 h at 37 °C. For checking the virucidal activity of conessine, PR8-GFP IAV and conessine were mixed and incubated for 30 min at 4 °C. The mixtures were added to the cells for 30 min at 37 °C and washed with PBS to remove the remaining virus. The cells were further incubated for 24 h at 37 °C. The levels of GFP expression were determined using fluorescence microscopy (Nikon Corp., Tokyo, Japan) with 200× magnification and FACS analysis.

### 4.8. Immunofluorescence Analysis

A549 cells were infected with a mixture of H1N1 IAV (10 MOI) and conessine (10 µM), preincubated for 1 h at 4 °C. After washing with PBS, the cells were further incubated for 24 h at 37 °C. The cells were fixed with 4% paraformaldehyde for 10 min, washed with PBS, and blocked with 1% BSA-containing PBS. After incubation with antibodies (GeneTex, Irvine, CA, USA) specific for influenza virus proteins for 12 h at 4 °C, the cells were incubated with Alexa Fluor 594 secondary antibody (Invitrogen, Waltham, MA, USA) for 1 h at 37 °C. After washing with PBS, Hoechst 33342 (Invitrogen, Waltham, MA, USA) was added for 5 min. The virus proteins and nuclei images were visualized and merged using fluorescent microscopy (Nikon Corp., Tokyo, Japan).

### 4.9. Hemagglutination Inhibition Analysis

The mixtures of conessine (1, 2, 5, or 10 µM) and 10 MOI H1N1 IAV were added to a 96-well U-bottom plate. An equal volume of 1% chicken Red Blood Cells (RBCs) (Innovative Research, Inc., Southfield, MI, USA) was added to each well and incubated for 1 h. RBCs in the absence of viruses were aggregated. RBCs in the virus-infected well were hemolyzed. HA titers were calculated as HA units/100 µL compared to the virus control.

### 4.10. Neuraminidase Inhibition Analysis

The effect of conessine on neuraminidase activity was examined using the NA-Fluor influenza Neuraminidase Assay Kit (Life Technologies, Carlsbad, CA, USA). Conessine was 2-fold serially diluted from 40 μM to 1.25 μM and mixed with H1N1 IAV in 96 well-black plates. Oseltamivir carboxylate (Aobious Inc., Gloucester, MA, USA), a specific NA inhibitor, was diluted from 10 μM to 0.01 μM and used as a positive control. As a recommendation of the instructor’s protocol, the neuraminidase activity was performed, and the values were determined using a fluorescence spectrometer (Promega, Madison, WI, USA) at an excitation wavelength of 365 nm and an emission wavelength of 445 nm.

## 5. Conclusions

Conessine potently inhibited influenza A virus infection by preventing viral binding and entry into the cells through the modulation of HA. Conessine eradicated the IAV before it could bind and enter the cells at the early time of infection. Our findings suggest that conessine could be used as a potential anti-influenza viral agent by combining it with late-stage inhibitors such as oseltamivir and zanamivir.

## Data Availability

All data related to this study are included in this article.
